# Sequence Evolution of the Intrinsically Disordered and Globular Domains of a Model Viral Oncoprotein

**DOI:** 10.1371/journal.pone.0047661

**Published:** 2012-10-31

**Authors:** Lucía B. Chemes, Juliana Glavina, Leonardo G. Alonso, Cristina Marino-Buslje, Gonzalo de Prat-Gay, Ignacio E. Sánchez

**Affiliations:** 1 Protein Structure-Function and Engineering Laboratory, Fundación Instituto Leloir and IIBBA-CONICET, Buenos Aires, Argentina; 2 Protein Physiology Laboratory, Departamento de Química Biológica, Facultad de Ciencias Exactas y Naturales, Universidad de Buenos Aires, Ciudad Universitaria, Buenos Aires, Argentina; 3 Structural Bioinformatics Laboratory. Fundación Instituto Leloir and IIBBA-CONICET, Buenos Aires, Argentina; Uni. of South Florida, United States of America

## Abstract

In the present work, we have used the papillomavirus E7 oncoprotein to pursue structure-function and evolutionary studies that take into account intrinsic disorder and the conformational diversity of globular domains. The intrinsically disordered (E7N) and globular (E7C) domains of E7 show similar degrees of conservation and co-evolution. We found that E7N can be described in terms of conserved and coevolving linear motifs separated by variable linkers, while sequence evolution of E7C is compatible with the known homodimeric structure yet suggests other activities for the domain. Within E7N, inter-residue relationships such as residue co-evolution and restricted intermotif distances map functional coupling and co-occurrence of linear motifs that evolve in a coordinate manner. Within E7C, additional cysteine residues proximal to the zinc-binding site may allow redox regulation of E7 function. Moreover, we describe a conserved binding site for disordered domains on the surface of E7C and suggest a putative target linear motif. Both homodimerization and peptide binding activities of E7C are also present in the distantly related host PHD domains, showing that these two proteins share not only structural homology but also functional similarities, and strengthening the view that they evolved from a common ancestor. Finally, we integrate the multiple activities and conformations of E7 into a hierarchy of structure-function relationships.

## Introduction

Many traditional concepts of protein science were originally developed for globular domains and are now challenged by intrinsically disordered domains [1]. Structure-function relationships in globular domains are often pictured in terms of a single average structure that harbors one or several catalytic or binding sites on its surface. On the other hand, disordered domains present multiple conformational states and protein function is instead traced to short sequences called “linear motifs” [2]. Linear motifs are usually assumed to depend on the presence of less than five function-determining residues and are considered as independent functional units [2]. As opposed to globular domains, there is no general consensus for the representation of structure- function relationships in disordered domains. Evolutionary structural biology commonly describes globular domains in terms of a continuous sequence alignment with a low percentage of gaps [3]. Conserved and coevolving residues within the core of a globular domain are assigned a structural role [4], while those on the surface are suggested to convey affinity and specificity for other molecules [3]. Currently, the relationship between conservation, co-evolution and function in disordered domains, also called “evolutionary unstructural biology” [5], is still unclear. For disordered regions, alignments are often unreliable [6] and contain a high percentage of gaps [7], [8]. Also, disordered domains are considered to present a lower degree of sequence conservation and co-evolution than globular domains [7], [8], [9] and have different amino acid substitution patterns [10], [11], [12]. Moreover, globular domains can also present linear motifs. The static protein paradigm is at odds with the presence of functional linear motifs because such motifs are believed to exert their function only within a context of structural disorder. Therefore, the well-known dynamic nature of proteins should also be considered for evolutionary models of globular domains containing linear motifs.

**Figure 1:**
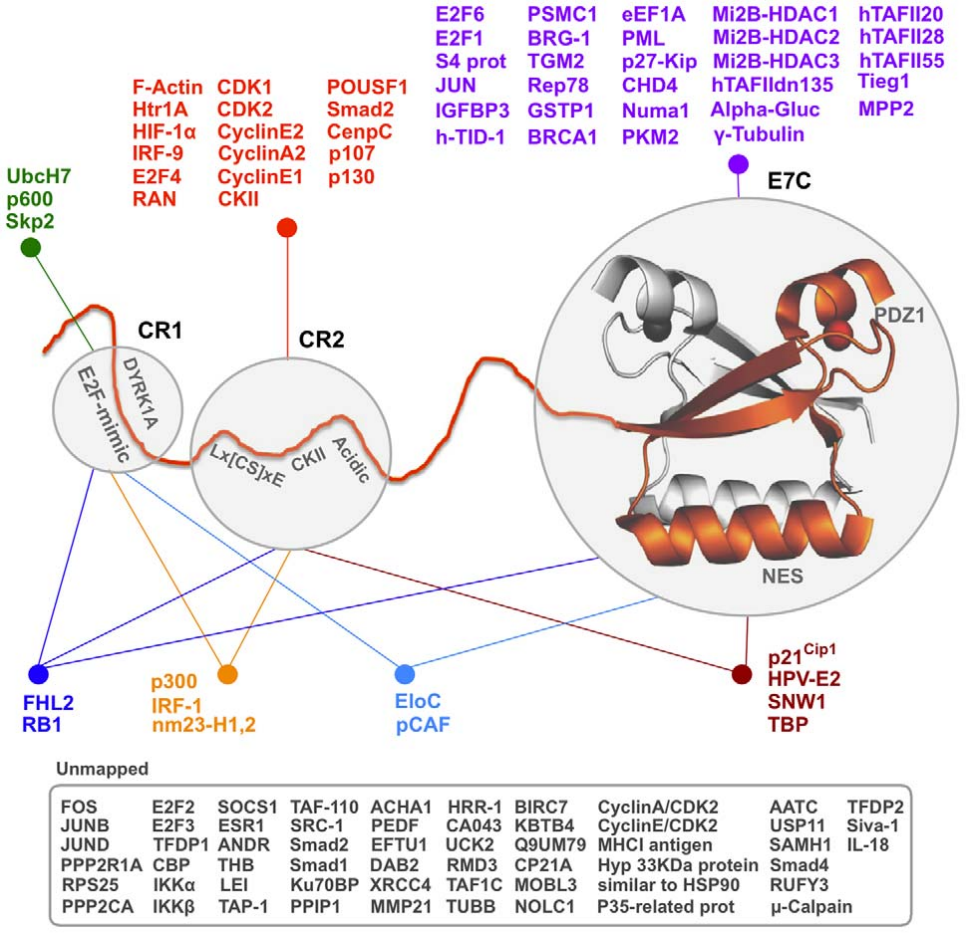
Schematic representation of the structure of the papillomavirus E7 protein and its protein targets. E7C is represented using the average NMR structure of the HPV45 E7C domain (PDB ID: 2F8B) and its associated zinc atoms as spheres. The E7N for one of the E7 monomers is represented in orange as an extended ribbon. The approximate locations of the CR1 and CR2 regions and of the E2F mimic, DYRK1A, Lx[CS]xE, CKII, acidic NES and PDZ motifs are also shown. E7 protein targets whose single or multiple binding sites have been mapped are shown grouped according to their interaction sites. Targets whose interaction sites have not been mapped are boxed.

The papillomavirus E7 protein is an interesting model system to compare sequence and function evolution in disordered and globular domains [13]. E7 contains a disordered N-terminal domain (E7N) formed by the conserved regions CR1 and CR2 and a globular homodimeric C-terminal domain (E7C) [14], [15], [16], [17] (Figure 1). The disordered E7N domain contains multiple functional linear motifs that mediate ubiquitination [18], phosphorylation by the DYRK1A [19] and caseine II [20] kinases, binding to the AB domain of the retinoblastoma protein at the E2F [21] and Lx[CS]xE sites [21], [22] and binding to the papillomavirus E2 master regulator [23]. The Lx[CS]xE motif and the CKII- Acidic region cooperate in Rb binding, showing that some of these linear motifs are functionally coupled [21], [22]. The globular E7C domain also presents two linear motifs, namely a nuclear export signal [24] and a PDZ binding motif [25]. These linear motifs can account, at least in part, for the high number of binding partners reported for E7 and for the multiple binding interfaces between E7 and some of its targets (Figure 1) [14], [15]. Some of the E7 targets, such as the retinoblastoma protein, also interact with multiple host proteins, leading to formation of a complex and poorly understood virus-host protein interaction network.

Furthermore, the globular E7C domain shows a range of conformations that may contribute to the protein interaction repertoire of E7. E7C is often regarded as a homodimerization and zinc-binding module [16], [17], but the dimer is not the only active conformation of E7C. For example, the transforming ability of E7 is unaffected by several mutations that impair dimer formation [26]. E7C monomers [14], [27] modulate binding to the AB domain of the retinoblastoma protein [21], [22] and may be able to bind zinc, while E7C large structured oligomers [27], [28] present a chaperone activity [29]. Last, the globular E7C domain can bind to an unstructured peptide from the host protein p21 [17], which suggests that E7C may also be a linear motif binding module. It has been proposed that the E7C fold arose from a host PHD domain [30], which is involved in protein-protein interactions. The published results deal only with structural homology, and the issue of functional similarity was not explored. Here, we have used the papillomavirus E7 protein to study structure-function relationships and sequence evolution of disordered and globular domains.

## Methods

### Sequence database

We retrieved all papillomavirus types in the NCBI taxonomy database as of June, 2011. 224 papillomavirus types had at least one ORF coding for an E7 protein ( File S1), except for *Tursiops truncatus papillomavirus types 1*, *2* and *3*, *Delphinus delphis papillomavirus*, *Sus scrofa papillomavirus type 1*, *Ursus maritimus papillomavirus type 1* and *Phocoena spinipinnis papillomavirus type 1*. Variant E7 sequences have been reported for many clinically important types, such as HPV16. Since our goal was to assess evolution of the E7 protein across papillomavirus types, we kept a balanced representation by retrieving a single E7 sequence for each type. Seventeen E7 sequences from reptilian [31], avian [32] and some artiodactyl [33] papillomaviruses had an N-terminal domain sequence with no recognizable homology to the other E7N domains. Five E7 sequences from chelonian and avian papillomaviruses presented a deletion of five to six residues in the C-terminal domain corresponding to the main alpha helix and are likely essential to maintain the known globular structure [34]. Thus, they were excluded from the sequence alignments.

### Sequence alignment

The remaining sequences were used to build separate E7N and E7C alignments, with 207 and 219 sequences respectively (Files S2 and S3). The software MUSCLE [35] was used to construct the initial alignments, using default parameters. The alignments were manually curated taking into account the known structures [16], [17] and functional sites [18], [19], [20], [21], [22], [23], [36]. Two additional degapped E7N and E7C alignments were produced by removing positions with more than 30% gaps (Files S4 and S5).

### Sequence conservation and co-evolution

Sequence logos [37] describing residue conservation were generated with WebLogo [38] and the degapped alignments of E7N and E7C. The information content *R(l)* for confidently aligned positions of the E7N and E7C domains (Files S4 and S5) was calculated as follows [37]:
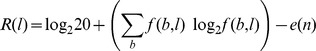
where 20 is the alphabet size for proteins, *f(b,l)* are the fractions of each amino acid *b* at position *l*. The third term is a small sample correction, where *n* is the number of sequences in the alignment. The maximum value of *R(l)* is 4.32, and the minimum is zero.

Mutual information (MI) describing the co-evolution of residue pairs was calculated as in [4]. Briefly, the MI is calculated between pairs of columns in the multiple sequence alignment. The frequency for each amino acid pair is compared to the expected pair frequency assuming that the amino acids are non-correlated. Next, the MI is calculated as a weighted sum of the log-ratios between the observed and expected amino acids pair frequencies. The APC method of Dunn et al. [39] was applied to reduce the background mutual information signal for each pair of positions and the MI scores were finally translated into MI Z-scores by comparing the MI values for each possible pair of positions to a large set of MI values calculated from permuted multiple sequence alignments.

### Motif discovery

We searched for putative binding motifs within the sequences of the proteins reported to interact with the E7C domain (File S6). We considered only the sequence segments reported to be necessary and sufficient for the interaction and discarded globular domains, where the likelihood of finding a functional linear motif is lower. We used two search algorithms. First, we used LeitMotif, an in-house implementation [40] of an algorithm previously developed to identify protein-binding sites from unaligned DNA sequences [41]. The algorithm performs a greedy search for short sequence alignments of high information content [41]. Second, we used DILIMOT, a server that extracts short, over-represented peptide patterns from protein-protein interaction datasets [42].

## Results

### Sequence evolution of the intrinsically disordered E7N domain

**Figure 2:**
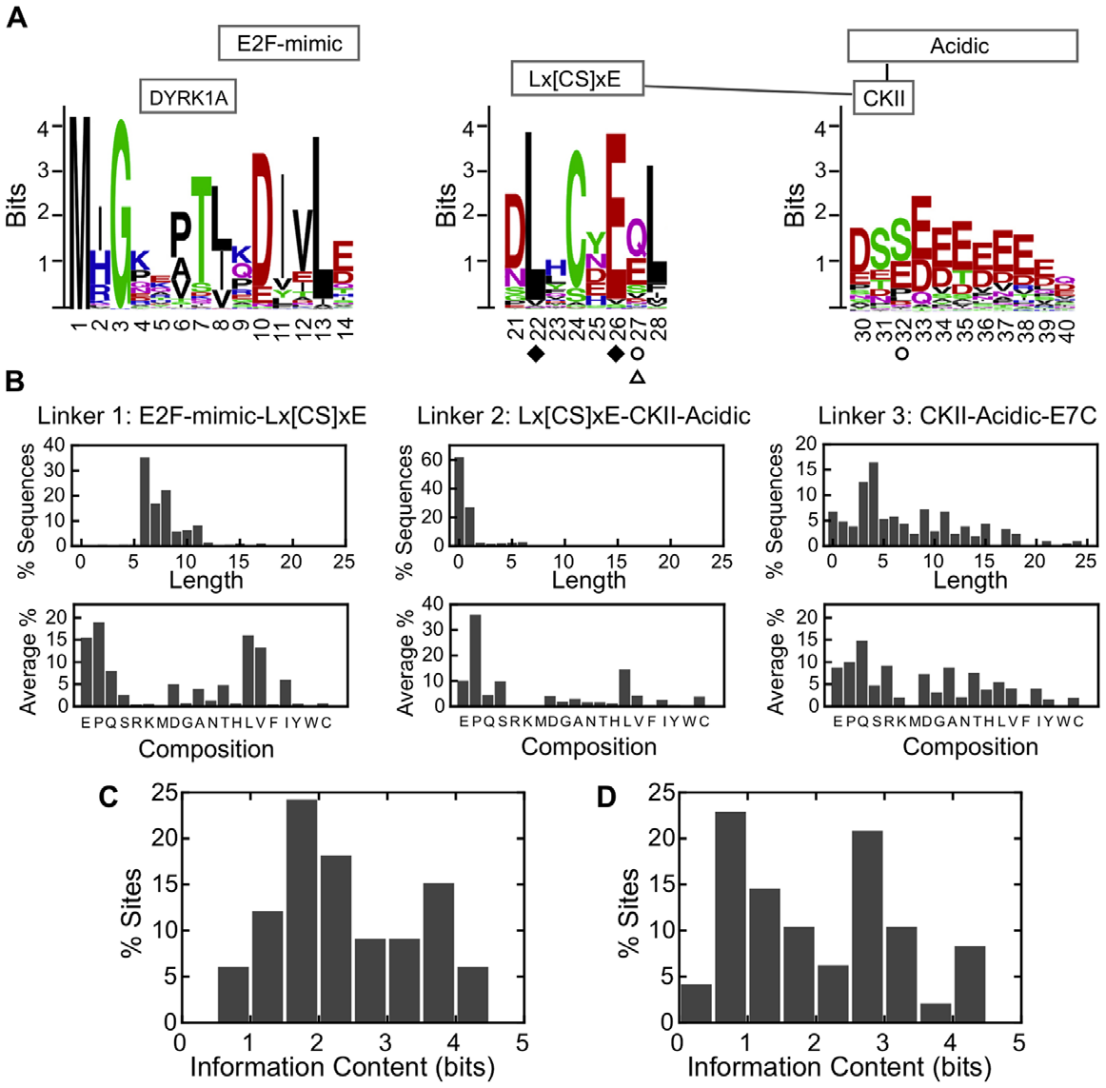
Sequence evolution of the intrinsically disordered E7N domain. (**A**) Graphical representation of sequence conservation and co-evolution in the E7N domain obtained from the alignment of 207 PV E7 sequences. Sequence stretches that could be aligned with confidence are depicted as sequence logos, and sequence numbering originates in the HPV16 E7 protein. The known functional motifs are shown above the logos as boxes and co-occurring motifs are joined by continuous lines [34]. Pairs of coevolving residues are indicated as filled diamonds, open circles and open triangles, respectively. (**B**) Highly divergent sequence stretches that could not be aligned with confidence are depicted as histograms of the length of each stretch (upper panel) and as average residue abundances (lower panel). Amino acids are ordered according to decreasing tendency to appear in disordered regions [10]. Linkers 1, 2 and 3 join positions 14 and 21, 28 and 30 and 40 and 51 in the E7 sequence logo, respectively. (**C**) Distribution of the information content of confidently aligned positions in the E7N domain (average is 2.4±1.0 bits). (**D**) Distribution of the information content of confidently aligned positions in the E7C domain (average is 2.1±1.2 bits).

**Figure 3:**
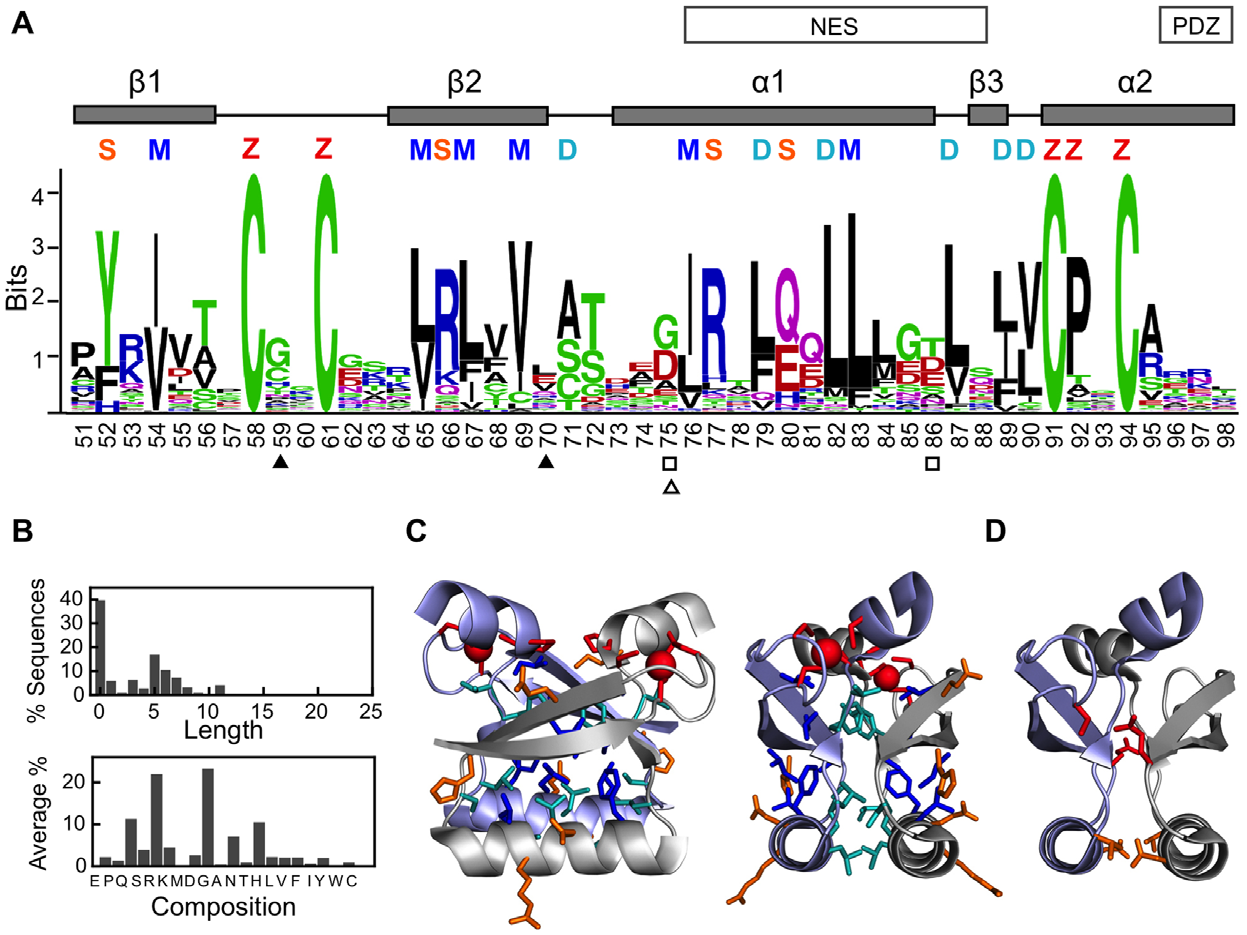
Sequence evolution of the globular homodimeric E7C domain. (**A**) Graphical representation of sequence conservation and co-evolution in the E7C domain, obtained from the alignment of 219 PV E7 sequences. Sequence numbering originates in the HPV16 E7 protein. The secondary structure of the E7C domain is depicted above the sequence. Highly conserved positions are marked by letters. These correspond to surface residues (S, orange), residues involved in the monomer hydrophobic core (M, blue), the dimerization interface (D, light blue) and residues involved in zinc binding (Z, red). Pairs of coevolving residues are indicated as filled triangles, open squares and open triangles, respectively. The locations of the known functional motifs are shown above the logos as boxes. The PDZ motif occurs at the C-terminus of each E7 protein but for clarity is shown at the end of the E7C sequence logo. (**B**) The C-terminus is depicted as a histogram for the length of the stretch (upper panel) and as average residue abundances (lower panel). Amino acids are ordered according to decreasing tendency to appear in disordered regions [10]. (**C**) Highly conserved E7C residues. Left and right views differ in a 90 degrees rotation. Residues mainly involved in the hydrophobic core of each monomer (M), the dimerization interface (D), zinc binding (Z), and highly conserved surface (S) residues are shown in stick representation according to the color coding of panel (A). (**D**) Representation of E7C coevolving residue pairs. The pair corresponding to residues 75 and 86 is shown in orange and that corresponding to residues 59 and 70 is shown in red. Protein representations use the average NMR structure of the HPV45 E7C domain (PDB ID: 2F8B). Protein representations were generated using Pymol (http://www.pymol.org).

We examined the previously reported sequence alignments of 207 E7N domains and 219 E7C domains [34]. The alignment of E7N sequences showed two types of clearly disparate regions. On one hand, several blocks of positions presented less than 30% gaps and were easily aligned. These regions could be pictured as sequence logos (Figure 2A). On the other hand, most positions outside these stretches presented more than 30% gaps and could not be aligned with confidence. These variable regions could be represented as a histogram of the observed length of the stretches and the average residue compositions (Figure 2B). Next, we examined and compared sequence conservation and co-evolution of the E7N and E7C domains. We used the information content of a position in the alignment as a measure of conservation [37], including all positions in the alignment with less than 30% gaps. Interestingly, the average conservation for the disordered E7N domain and the globular E7C was very similar (E7N 2.4±1.0 bits, E7C 2.1±1.2 bits) (Figures 2C and 2D). An algorithm based on mutual information [4] identified two pairs of coevolving residues within the E7N domain (Figure 2A) and two pairs of coevolving residues within the E7C domain (Figure 3A). These results indicated that a lack of globular structure did not necessarily lead to a lower degree of sequence conservation and co-evolution in the E7 protein. Co-evolution of one E7N residue with a residue on the surface of the E7C domain (Figure 2A and 3A) suggested that some relevant activity of the protein may involve physical contact between the domains and that evolution of the two domains in E7 had not been entirely independent.

Most conserved and confident aligned E7N stretches corresponded to well-known functional sites, such as the CR1 ubiquitination site at the N-terminus, the E2F mimic and the DYRK1A sites and the CR2 Lx[CS]xE motif, CKII sites and acidic stretch. The sequence logos showed high information content (*R(l)*>2.0) for many positions. The most conserved position in the N-terminus ubiquitination site, in addition to the N-terminal methionine, was residue 3. The DYRK1A site (residues 5–7, Figure 2A) is only partially conserved. All 7 positions of the E2F mimic (residues 8–14, Figure 2A) showed an intermediate-to-high degree of conservation. Within the Lx[CS]xE motif (residues 22–26, Figure 2A) two extra positions, 21 and 28 were also highly conserved. Positions 22 and 26 within the Lx[CS]xE motif coevolved, consistent with the motif being a functional unit. Co-evolution of position 27 in the Lx[CS]xE motif and position 32 in the CKII-acidic region suggested that these two regions evolved in a coordinate manner. The length and composition of the different sequence segments connecting the known sites showed variability (Figure 2B). At one extreme, there was no linker between the N-terminal ubiquitination site, the DYRK1A site and the E2F mimic. We did observe a linker between the E2F mimic and the Lx[CS]xE motif, with a length of 7.7±2.1 residues and a composition that favored proline, valine, leucine and glutamate residues. The distance between the Lx[CS]xE motif and the CKII-acidic region was short and highly restricted (0.7±1.4 residues), and the linker was rich in proline residues. Last, the distance from the CKII-acidic region and the C-terminal domain of E7 was both the longest and had the widest range (7.2±5.4 residues). In addition, it was rich in disorder-promoting residues [10]. Interestingly, those E7N motifs with coevolving residues and joined by a restricted linker, namely the Lx[CS]xE and CKII-Acidic motifs, were previously identified as co-occurring motifs [34].

### Sequence evolution of the globular homodimeric E7C domain

The alignment of E7C sequences showed a very good average quality and a small percentage of gaps over the region with a globular homodimeric structure in HPV1a and HPV45 E7 proteins [16]–[17], which we pictured as a single sequence logo (Figure 3A). Only in the C-terminal positions there was a high percentage of gaps and potential ambiguities. We represented the variable C-terminus with histograms of the observed length and average residue composition (Figure 3B).

Several groups of highly conserved residues (*R(l)*>2.0) could be readily identified on the structure of the E7C homodimer (Figure 3C). First, four cysteines and a proline are the binding site for the zinc atom (red, Figure 3C). Second, six residues constitute the hydrophobic core of each monomer (blue, Figure 3C). Third, six residues form the hydrophobic dimerization interface (light blue, Figure 3C). Additionally, four surface residues (positions 52, 66, 77 and 80, orange) were also highly conserved (orange, Figure 3C). There were two pairs of coevolving residues in the E7C domain, 59/70 and 75/86 (Figure 3A, triangles and squares respectively), both of which form close contacts across the dimerization interface (Figure 3D, red and orange respectively). Thus, there was a good correspondence between the conservation and co-evolution patterns and the homodimer structure. This conclusion was in agreement with a recently published mutagenesis study on HPV16 E7C dimerization [26]. Out of the 12 residues reported by this work to be important for dimerization, we identified 10 to be highly conserved (positions 65, 67, 69, 76, 79, 87, 89/90 and 91) or coevolving (position 86), while only surface positions 57 and 84 showed low conservation. Two linear motifs are located in the globular E7C domain, the nuclear export signal formed by five residues from the monomer and dimer cores (NES, residues 76–89 Figure 3A) and a PDZ binding motif (PDZ) located at the C-terminus. We found that while the nuclear export signal was highly conserved, the PDZ motif was not conserved to a recognizable degree.

**Figure 4:**
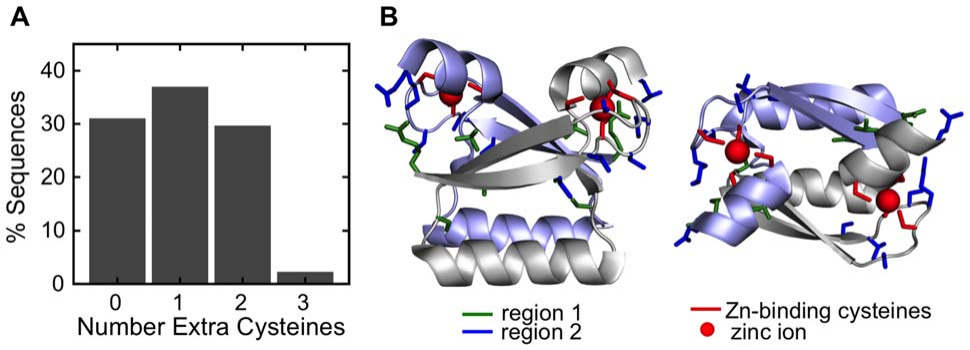
Cysteine-rich regions in the E7C domain. (**A**) Distribution of the number of cysteines in the cysteine-rich regions of individual E7C domains. (**B**) Ribbon representation of the average NMR structure of HPV45 E7C (PDB ID: 2F8B), with cysteine-rich positions corresponding to regions 1 (green) and 2 (blue) in stick representation (see text). Note that for many cysteine-rich positions the corresponding HPV45 E7C residue is not a cysteine. Zinc atoms are represented as red spheres. Protein representations were generated using Pymol (http://www.pymol.org).

Visual inspection of the E7C alignment in the vicinity of the zinc-binding CxxC motifs suggested the presence of an unusually high number of cysteine residues. Ten positions of the alignment other than the canonical cysteines indeed presented at least 5.9% of Cys, four-fold higher than the average percentage of cysteines in Uniprot, 1.36% [43]. About 70% of E7C domains had at least one extra, non-canonical cysteine residue (Figure 4A). Out of these, two thirds had one extra cysteine and one third had two extra cysteines, with only 3% having three extra cysteines (Figure 4A). These non-canonical cysteine-rich positions could be classified in two clusters. One of them included positions 56 (6.8%), 57 (10.5%), 59 (18.7%), 60 (9.4%), 63 (5.9%) and 98 (9.7%), which are close in sequence and space to the CxxC motif of each E7C monomer (blue residues in Figure 4B). The second cluster included positions 51 (8.3%), 68 (9.1%), 69 (6.8%) and 71 (20.1%). These residues were farther away in sequence from the CxxC motifs, but the cluster of one E7C monomer was close in space to the CxxC motif of the other molecule in the homodimer (green residues in Figure 4B). These results suggest a yet undescribed functional role for these residues.

### A recognition site for linear motifs in the E7C domain

**Figure 5:**
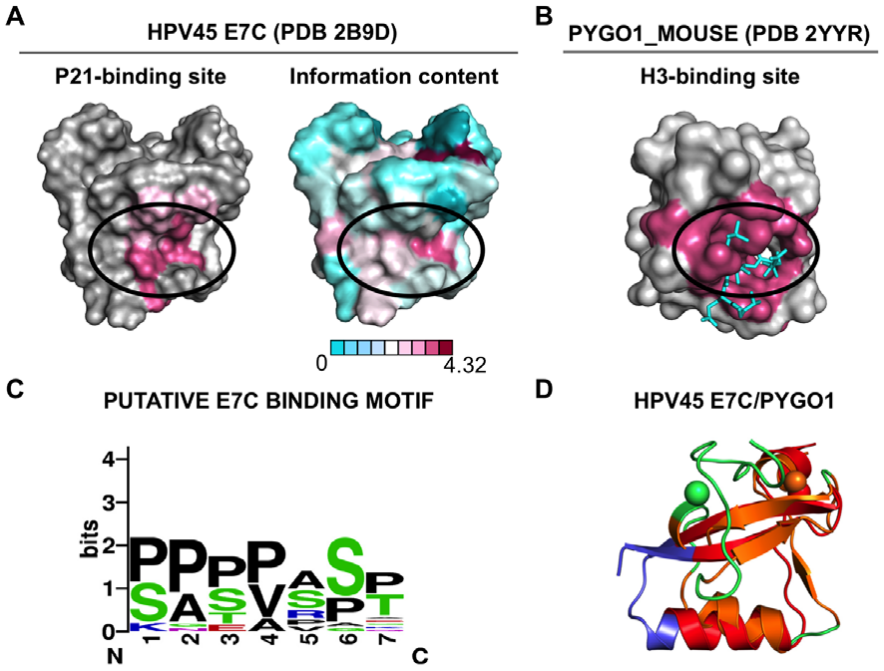
A recognition site for linear motifs in the E7C domain. (**A**) Left: Binding site for the p21 protein [17], represented on the surface of the average NMR structure of HPV45 E7C (PDB ID: 2F8B). The ellipse highlights residues whose amides are strongly (dark pink) or moderately (light pink) perturbed by binding. Right: Conservation of surface E7C residues, represented on the surface of the average NMR structure of HPV45 E7C (PDB ID: 2F8B). Conservation of a residue is measured as information content and binned in nine categories (at 0.5 bits intervals from 0 to 4, with an extra interval from 4 to 4.32 bits). The ellipse highlights a conserved surface patch. (**B**) Complex between the PHD domain of Pygopus (PDB ID: 2YYR, in surface representation) and a trimethylated histone H3 peptide (light blue, sticks representation). The ellipse highlights residues in direct contact with the peptide (dark pink). The PHD domain is oriented as in (D). (**C**) Sequence logo representation of the putative E7C target linear motif obtained by LeitMotif. (**D**) Structural alignment of the HPV45 E7C monomer (blue) and the PHD domain of Pygopus (green). Structurally aligned regions from E7C and the PHD domain are colored in red and orange respectively. The E7C monomer is oriented as in (A), and the PHD domain is oriented as in (B). Protein representations were generated using Pymol (http://www.pymol.org).

The surface of E7C has been mapped as the interaction site for an unstructured peptide from the host protein p21 [17] (Figure 5A, left), the unstructured RbC domain [16] and an unstructured domain within the Mi2β protein [44]. This suggested that E7C could bind to linear motifs contained within disordered domains of its targets. We have plotted the information content of each alignment position on a surface representation of the E7C homodimer (Figure 5A, right). The data showed a moderately conserved patch on the surface of E7C, which partially overlapped with the reported p21 binding surface. These results suggested that a significant fraction of E7C domains may interact with protein p21 at this site, and that this may be the interaction site with other targets.

More than 35 proteins have been reported to interact with the E7C domain (Figure 1 and Figure S1) but for most of these targets the interaction site has not been mapped. We hypothesized that a fraction of these targets may share a linear motif that mediates interaction with the p21 interaction surface (Figure 5A). We have searched for such a motif within the E7C targets using two different algorithms: DILIMOT, which describes linear motifs as regular expressions [42] and LeitMotif, which describes them in a probabilistic manner [40]. Both methods yielded a set of high-scoring motifs rich in serine and proline residues and in Ser-Pro dipeptides (Figure S2). A representative example extracted using LeitMotif is shown in Figure 5C. Based on these results, we suggest that a serine-proline rich motif may be responsible for the interaction between several E7C targets and the p21 binding surface in E7C.

### Functional similarities between the E7C domain and distantly related host proteins

The E7C domain fold shows significant structural similarity with chromatin-remodeling proteins with the treble cleft fold [30]. The protein family with the highest similarity to the E7C monomer is the PHD domain. Figure 5D shows a representative structural alignment [45] of the HPV45 E7C monomer (PDB 2f8b) with the Pygopus PHD domain [46], [47] (PDB 2yyr), which spans all secondary structure elements of the E7C monomer. Remarkably, the Pygopus PHD domain surface equivalent to the putative binding site for linear motifs in E7C is able to bind methylated histone peptides [46], [47] (Figure 5B). This binding activity is also displayed by other PHD domains [48]. The PHD domain surface equivalent to the dimerization interface of E7C mediates formation of regulatory complexes. In the case of the MLL1 PHD domain, this surface binds with similar affinities to both the bromodomain in the same protein and the RRM domain in the Cyp33 protein [49]. In the case of the Pygopus PHD domain, this surface binds to the BCL9 HD1 domain [46], [47] and mediates formation of a PHD homodimer with a Kd of 1 µM [50]. Although this K_d_ is similar to that of the E7C homodimer [51], the orientation of the monomers in the Pygopus homodimer [50] differs from the orientation of the monomers in the E7C homodimer. Altogether, these facts suggest that E7 shares not only structural homology but also functional similarities with PHD domains, strengthening the view that they evolved from a common ancestor.

## Discussion

Our representation of the intrinsically disordered E7N domain (Figure 2) consists of a one-dimensional array of several short sequence alignments separated by unaligned linkers of varying lengths, reminiscent of the description of promoters as linear maps of transcription factor binding sites [52] and to the recently proposed concepts of “constrained disorder” and “flexible disorder” [53]. We have also added a coarse mapping of inter-residue relationships using residue co-evolution, restricted intermotif distances and motif co-ocurrence [34]. For the E7 oncoprotein we found that these signals provided useful tools for mapping functional coupling of linear motifs within its disordered domain, further indicating that E7N is a proper domain and not a mere juxtaposition of independent linear motifs [15].

Remarkably, the confidently aligned positions of the E7N domain are on average as conserved as the globular E7C domain and the number of coevolving residue pairs is the same (Figures 2 and 3). This is unusual for a disordered domain [7], [8], [9] and may stem from the high functional density in E7N, characteristic of many viral proteins [54]. Many of the conserved residues are those that determine the known motifs, such as the L, [CS] and E in Lx[CS]xE. Conserved position 3 has not been assigned to a motif, but may play a role in the unusual ubiquitination of E7, which is targeted to the N-terminus of the molecule [18]. Additionally, positions 21, 27 and 28 flanking the Lx[CS]xE motif and internal position 25 show significant conservation. Site-directed mutagenesis suggests that these non-canonical internal and flanking positions [11] are conserved because they contribute to strong target binding [55], [56]. Conservation may also be due to their role in modulating E7N conformational ensemble, which is far from random [1], [15].

Several E7N regions evolve in a coordinate manner (Figure 2), as shown by the coevolving residue pairs, the restricted linker lengths and motif co-occurrence [34]. On the other hand, consecutive E7N functional motifs are close in space to each other as indicated by the short linker lengths, while the co-evolution signals may indicate functional coupling or at least transient physical contact between the Lx[CS]xE motif and the CKII-Acidic region [4]. This physical contact can lead to the coupling of motif conformations, as observed for the coupling between the helix-coil and polyproline type II-coil transitions in E7N [15] and in the papillomavirus E2 protein [57], [58]. We propose that the molecular property conserved through coordinate evolution is the complex conformational behavior of E7N, which is likely linked to its multiple binding activities [15].

The analysis of conservation and co-evolution (Figure 3) shows that the known E7C homodimeric structure is relevant in evolutionary terms, in agreement with mutagenesis data [26]. The distantly related host PHD domains are also able to dimerize, suggesting that the ancestral E7C domain was also a homodimer. On the other hand, the dimer is incompatible with some of the known E7C activities. For example, chaperone activity depends on the formation of large oligomers [29]. Also, the residues that constitute the NES in E7C are buried in the structure of the homodimer and therefore not accessible to the CRM1 exportin. The same residues would be significantly exposed in the monomer, even more so if it loses structure upon dissociation (Figure S2). The monomer-dimer equilibrium may thus regulate the accessibility of the nuclear export signal [59] and consequently the relative populations of nuclear and cytoplasmic E7 molecules [27]. The micromolar dissociation constant of the dimer [51] fits well in this scenario.

Our results suggest that the E7C domain has other activities in addition to being a dimerization sequence. We report non-canonical cysteines in E7C, located on the surface of the homodimer and close in structure to the zinc-coordinating cysteines (Figure 4). Cysteine residues are usually involved in catalysis, metal coordination and constitute the main target for redox-regulation in proteins due to the reactivity of the thiol group. The E7C extra cysteines could play a role in redox regulation of E7 structure and function, as observed for many other proteins [60], including the papillomavirus L1 major capsid protein [61]. A common mechanism of virus-host interactions is the targeting of host linear motifs through a globular domain acquired from the host [54]. In agreement with this, we find that E7C is likely to bind sequences rich in serine and proline residues (Figure 5), akin to phosphorylation sites for cyclin dependent kinases, or CDKs [62]. We speculate that the E7C domain helps manipulate the cell cycle of the host cell by binding to CDK motifs [54].

**Figure 6:**
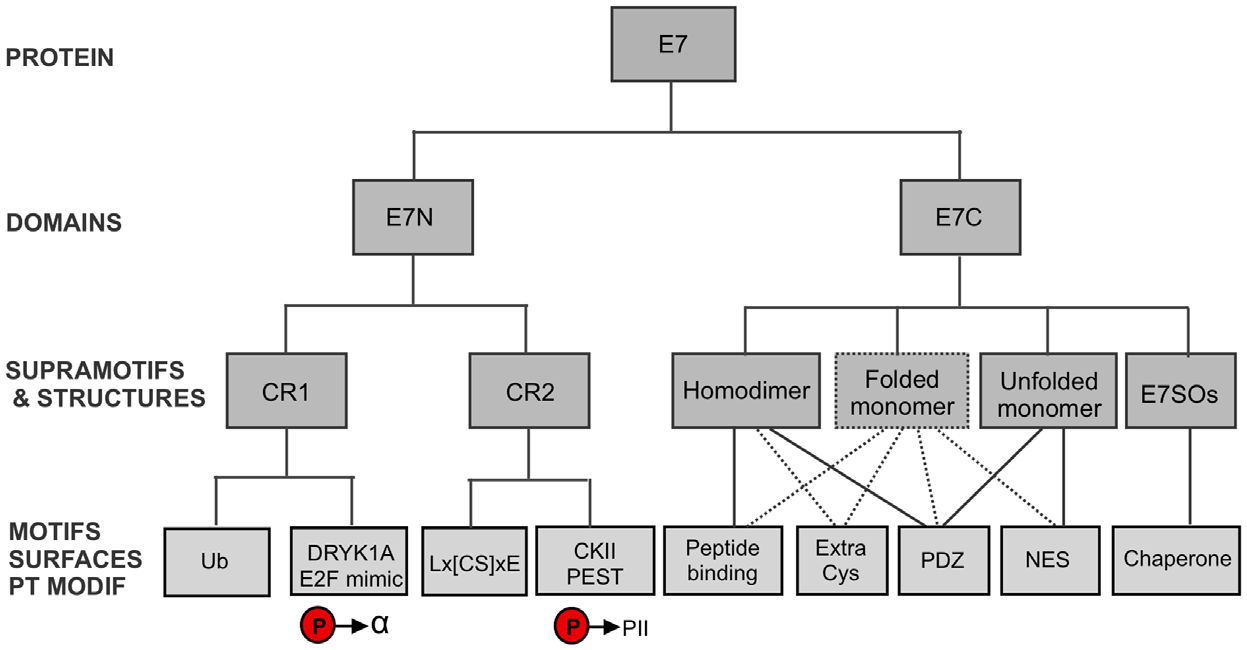
Structure-function relationships in the papillomavirus E7 protein. The bottom layer includes simple units that can be assigned a conformational transition or biological activity, including linear motifs and interaction surfaces. The second layer joins elements of the first layer that function and/or evolve together and the different structures where a biological activity is present (continuous line) or postulated (dashed line). The third layer assembles elements of the second layer into protein domains, i.e., longer sequence stretches that are structural and genetic units. The top layer integrates the two domains and is constituted by the E7 protein as a whole.

As a result of this work, we may draw an integrated, multi-layer scheme of E7 structure-function relationships (Figure 6). At the bottom layer we consider simple units that can be assigned a physicochemical or biological activity, such as linear motifs. We also list the peptide binding and chaperone activities of the E7C domain and its extra cysteines. At the next layer we join elements of the bottom layer that function and/or evolve together. For example, the Lx[CS]xE and the CKII-acidic motifs are located at a restricted distance, some of their residues coevolve and appear and disappear in a coordinate manner. We also consider known and putative structures, such as the E7C homodimer, folded monomer, unfolded state and large spherical oligomers. Pairs of elements from the first layer may be active or inactive in a given structure, such as the NES being inactive in the E7C homodimer. At the third layer we consider the E7N and E7C domains. These longer sequence stretches integrate several elements from the lower layers but are best understood as domains in structural and genetic terms [31], [32], [33]. Finally, our co-evolution data and the cooperation in the binding of retinoblastoma and several other cellular targets (Figure 1) support the view that the two domains function and evolve in a coordinate manner. Furthermore, the E7C domain turns E7 into a bivalent protein with two E7N domains. To sum up, we must consider a fourth layer with a single element, the E7 protein as a whole. The integration of multiple conformations, functional motifs and binding targets within E7 is likely related to the alteration of the cell cycle to the virus' benefit and may be linked to the development of cervical cancer. We envision that sequence-structure-function analyses similar to the one presented here may help our understanding of other viral oncoproteins and are a tentative step in “evolutionary unstructural biology” [5].

## Supporting Information

File S1 **List of E7 sequences included in the E7 sequence database.**
(XLS)

File S2 **E7N domain alignment with gaps.**
(TXT)

File S3 **E7C domain alignment with gaps.**
(TXT)

File S4 **E7N domain alignment without gaps.**
(TXT)

File S5 **E7C domain alignment without gaps.**
(TXT)

File S6 **Fasta sequences of the proteins reported to interact with the E7C domain.** We considered sequence segments reported to be necessary and sufficient for the interaction and discarded globular domains.
(TXT)

Figure S1 **High Scoring E7C peptide binding motifs.** A) Motifs obtained by LeitMotif shown as sequence logos. B) Motifs obtained by Dilimot shown as consensus sequences.
(TIF)

Figure S2 **Location of the residues that constitute the nuclear export signal (NES) in E7C.** Frontal (A) and side (B) views of the E7C homodimer (PDB ID 2F8B) showing the side chains of residues that form the NES signal (stick representation). Most residues are buried in the structure of the homodimer and located in the dimerization interface. These residues would be significantly exposed in the monomer. The cysteine residues and coordinated Zinc atoms are shown as reference.
(TIF)
