# Supplementary figures and images for: Sequence Evolution of the Intrinsically Disordered and Globular Domains of a Model Viral Oncoprotein
Source: PLoS One. 2012 Oct 31;7(10):e47661. doi: 10.1371/journal.pone.0047661 (PMC3485249; doi:10.1371/journal.pone.0047661)

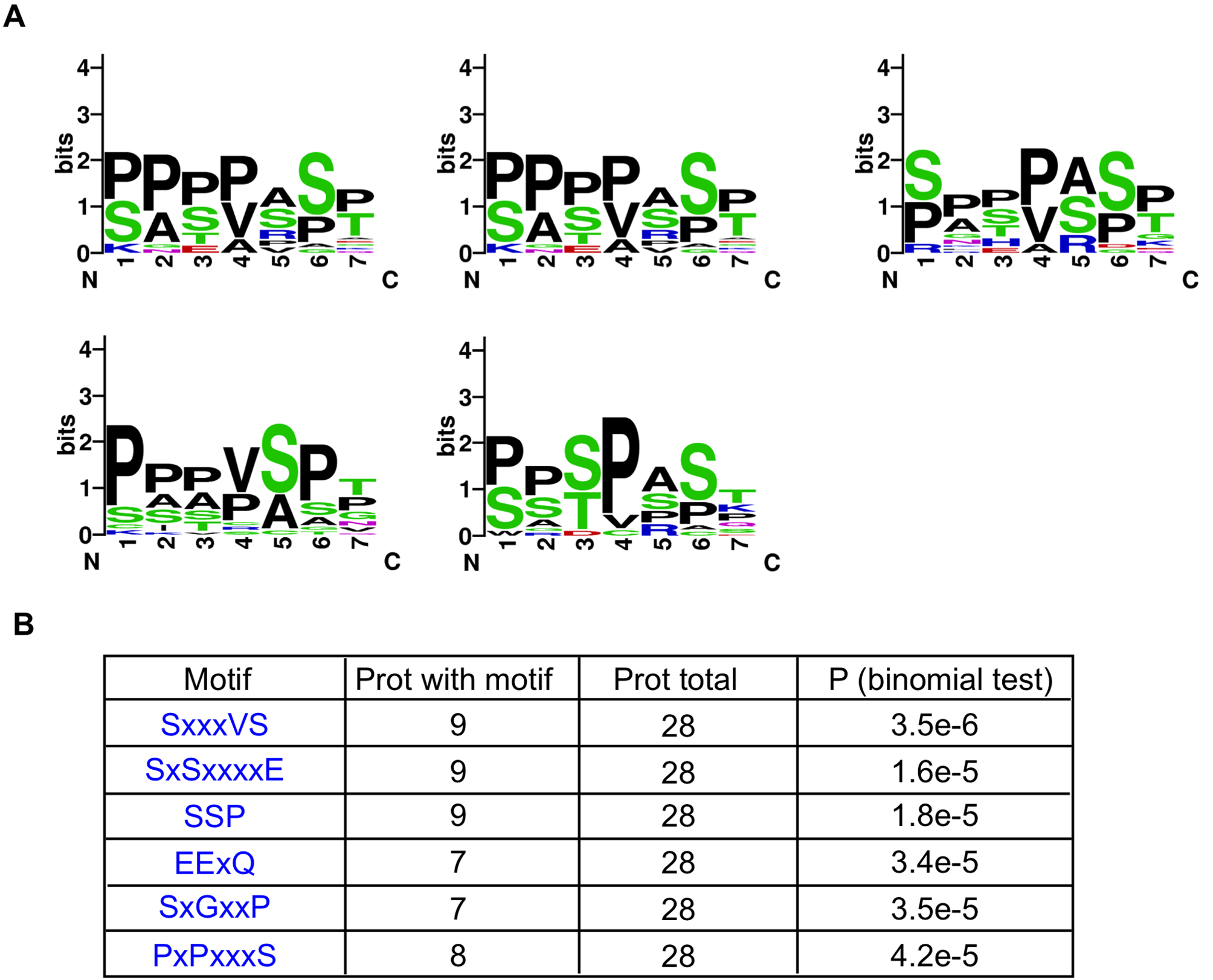

Supplement: Figure S1 — High Scoring E7C peptide binding motifs. A) Motifs obtained by LeitMotif shown as sequence logos. B) Motifs obtained by Dilimot shown as consensus sequences. (TIF) [file pone.0047661.s007.tif]

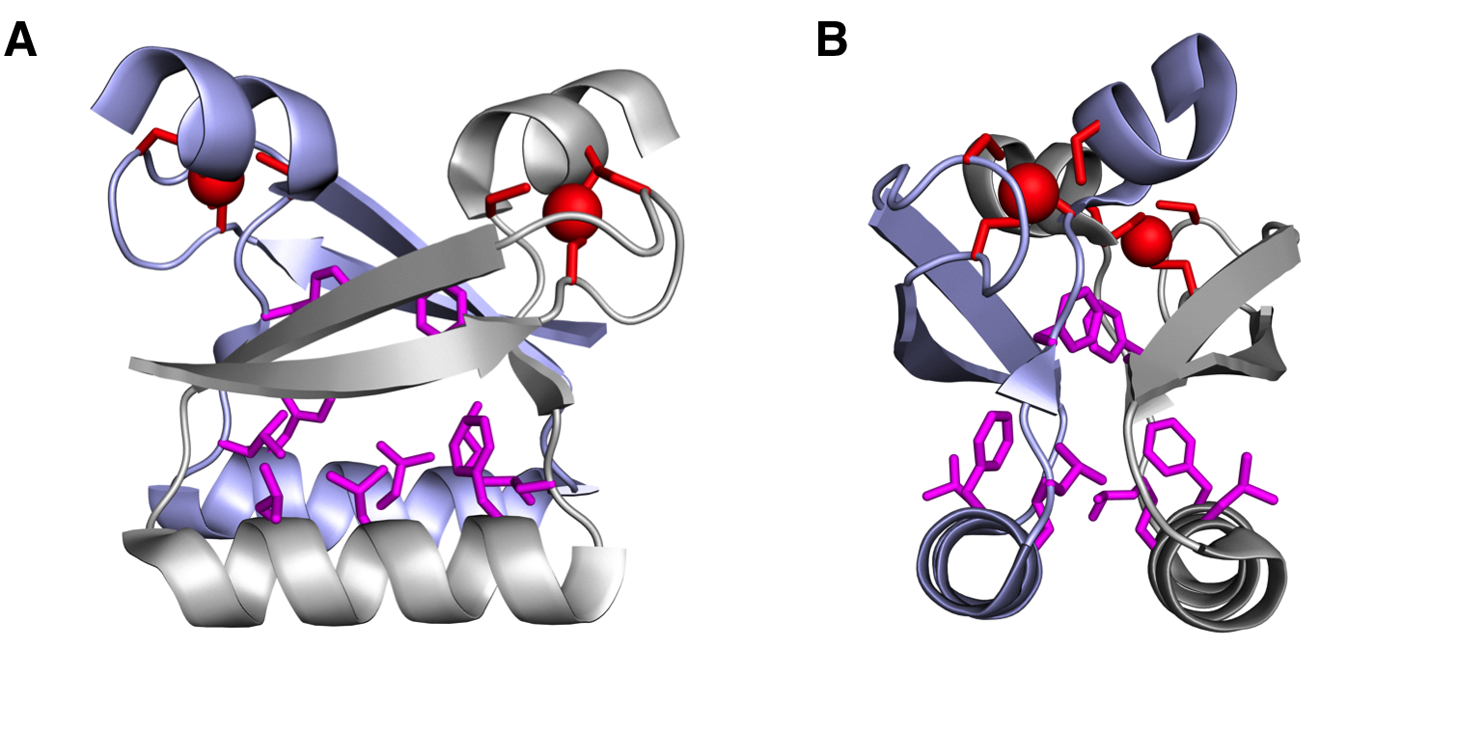

Supplement: Figure S2 — Location of the residues that constitute the nuclear export signal (NES) in E7C. Frontal (A) and side (B) views of the E7C homodimer (PDB ID 2F8B) showing the side chains of residues that form the NES signal (stick representation). Most residues are buried in the structure of the homodimer and located in the dimerization interface. These residues would be significantly exposed in the monomer. The cysteine residues and coordinated Zinc atoms are shown as reference. (TIF) [file pone.0047661.s008.tif]
